# (*E*)-5-(4-Meth­oxy­phen­yl)-*N*′-(2-oxoindolin-3-yl­idene)-1-phenyl-1*H*-pyrazole-3-carbohydrazide

**DOI:** 10.1107/S2414314623004558

**Published:** 2023-05-26

**Authors:** Bakr F. Abdel-Wahab, Abdelbasset A. Farahat, Mohamed S. Bekheit, Emad Yousif, Benson M. Kariuki, Gamal A. El-Hiti

**Affiliations:** a Applied Organic Chemistry Department, Chemical Industries Research Institute, National Research Centre, Dokki, Giza 12622, Egypt; b Master of Pharmaceutical Sciences Program, California Northstate University, Elk Grove, CA 95757, USA; c Department of Pharmaceutical Organic Chemistry, Faculty of Pharmacy, Mansoura University, Mansoura 35516, Egypt; d Department of Pesticide Chemistry, National Research Centre, Dokki, Giza 12622, Egypt; e Department of Chemistry, College of Science, Al-Nahrain University, Baghdad 64021, Iraq; f School of Chemistry, Cardiff University, Main Building, Park Place, Cardiff CF10 3AT, United Kingdom; g Department of Optometry, College of Applied Medical Sciences, King Saud University, Riyadh 11433, Saudi Arabia; University of Aberdeen, United Kingdom

**Keywords:** crystal structure, pyrazoles, carbohydrazides, isatin, heterocycles, synthesis

## Abstract

The asymmetric unit of the title compound, C_25_H_19_N_5_O_3_, is composed of two independent mol­ecules with slightly different conformations. The extended structure features N—H⋯O hydrogen bonds as well as π–π inter­actions.

## Abstract 2

The asymmetric unit of the title compound consists of two mol­ecules with slightly different conformations.

## Graphical abstract

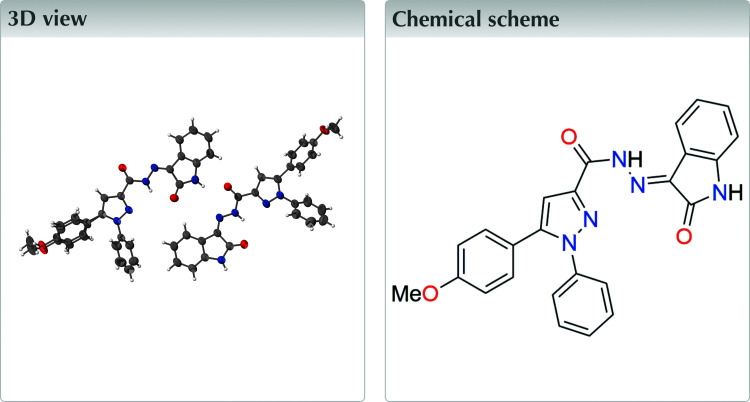

## Structure description

The synthesis of hydrazides and hydrazones is of inter­est because of their involvement in various synthetic transformations. In addition, they have potential pharmacological applications (see, for example, Afsah *et al.*, 2016 ▸). Isatin-containing heterocycles also exhibit biological activities, such as anti­convulsant, anti­microbial and anti­oxidant properties (see, for example, Kaur *et al.*, 2016 ▸). The most common synthetic methods for isatins involve the oxidation of indoles (Zi *et al.*, 2014 ▸), oxindoles (Wei *et al.*, 2017 ▸; Ying *et al.*, 2018 ▸) and amino­aceto­phenones (Qian *et al.*, 2017 ▸). Pyrazoles have several medicinal uses and can act as analgesics and anti­pyretics (see, for example, Rios & Portilla, 2022 ▸). Many synthetic procedures have been reported for the synthesis of pyrazoles (see, for example, Zora *et al.*, 2011 ▸; Karrouchi *et al.*, 2018 ▸; Li *et al.*, 2021 ▸). Recently, we have been investigating the synthesis and structure elucidation of various heterocycles. The current work describes the synthesis and the crystal structure of a hydrazone containing both isatin and pyrazole moieties using a simple procedure.

**Figure 1:**
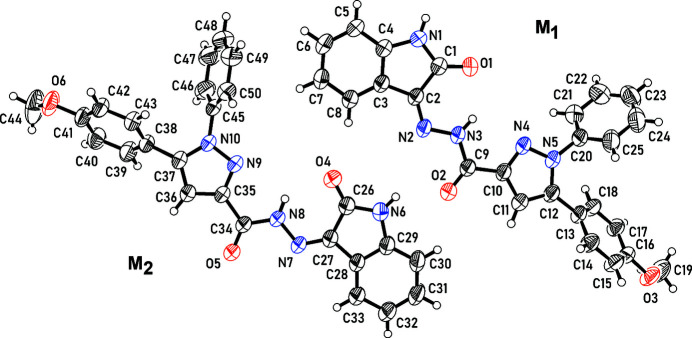
An *ORTEP* representation of the asymmetric unit of the title compound showing 50% probability atomic displacement parameters. Only the major disorder component of the disordered meth­oxy­benzene group of mol­ecule *M*_2_ is shown.

The crystal structure of the title compound, C_25_H_19_N_5_O_3_, contains two independent mol­ecules, *M*_1_ and *M*_2_ (Fig. 1 ▸). Each mol­ecule comprises indole-2-one (*M*_1_ *A*: C1–C8, N1, O1 and *M*_2_ *A*: C26–C33, N6, O4), formohydrazide (*M*_1_B: C9, N2, N3, O2 and *M*_2_B: C34, N7, N8, O5), pyrazole (*M*_1_ *C*: C10–C12, N4, N5 and *M*_2_ *C*: C35–C37, N9, N10), meth­oxy­benzene (*M*_1_ *D*: C13–C19, O3 and *M*_2_ *D*: C38–C44, O6) and phenyl (*M*_1_ *E*: C20–C25 and *M*_2_ *E*: C45–C50) moieties.

In both mol­ecules, the indole-2-one, formohydrazide and pyrazole groups are close to coplanar: the twist angles *M*_1_ *A*/*M*_1_ *B* and *M*_1_ *B*/*M*_1_ *C* are 5.0 (1) and 10.7 (1)°, respectively, for mol­ecule *M*_1_ and the corresponding angles *M*_2_ *A*/*M*_2_ *B* and *M*_2_ *B*/*M*_2_ *C* for mol­ecule *M*_2_ are 4.1 (2) and 9.7 (2)°, respectively. The near coplanarity of the indole-2-one and formohydrazide groups is similar to that observed for the related 1-(4-meth­oxy­phen­yl)-5-methyl-*N′*-(2-oxoindolin-3-yl­idene)-1*H*-1,2,3-triazole-4-carbohydrazide, **3** (Kariuki *et al.*, 2022 ▸) and 1-(4-fluoro­phen­yl)-*N′*-(2-oxoindolin-3-yl­idene)-5-phenyl-1*H*-1,2,3-triazole-4-carbohydrazide, **4** (Mohamed *et al.*, 2023 ▸).

The meth­oxy­benzene and phenyl groups deviate from the plane of the indole-2-one and formohydrazide groups. The twist angles *M*_1_ *C*/*M*_1_ *D* and *M*_1_ *C*/*M*_1_ *E* are 51.9 (2) and 55.8 (1)°, respectively, for mol­ecule *M*_1_ while *M*_2_ *C*/*M*_2_ *D* and *M*_2_ *C*/*M*_2_ *E* are 51.7 (2) and 57.7 (1)°, respectively, for mol­ecule *M*_2_. The twists of the two aromatic rings from the plane of the pyrazole group is of the same order as observed in **3** (Mohamed *et al.*, 2023 ▸) as well as in 4-{3-[2-(4-meth­oxy­benzyl­idene)hydrazine-1-carbon­yl]-5-(4-meth­oxy­phen­yl)-1*H*-pyrazol-1-yl}benzene­sulfonamide and 4-{5-(4-bromo­phen­yl)-3-[2-(4-methyl­benzyl­idene)hydrazine-1-carbon­yl]-1*H*-pyrazol-1-yl}benzene­sulfonamide, **5** (Lu *et al.*, 2016 ▸). Intra­molecular N—H⋯O hydrogen bonds (Table 1 ▸) contribute to the observed mol­ecular planarity. Similar intra­molecular N—H⋯O hydrogen bonds also occur in **3** and **4**.

**Figure 2:**
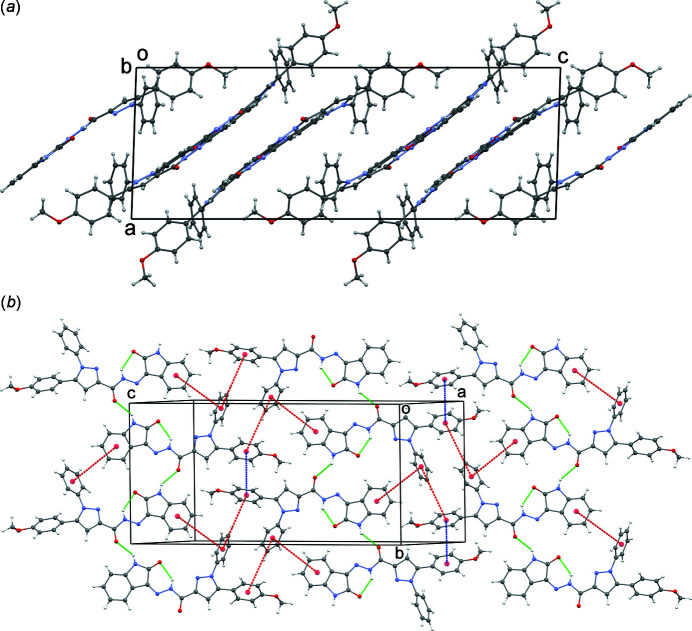
(*a*) Crystal packing for the title compound and (*b*) a layer showing the edge-to-face inter­actions (red dotted lines), π–π inter­actions (blue) and N—H⋯O hydrogen bonds (green).

In the extended structure of the title compound, the mol­ecules form layers oriented parallel to (102) (Fig. 2 ▸ *a*). Inter­molecular N—H⋯O hydrogen bonds (Table 1 ▸) occur within the layers. In the layers, aromatic ring edge-to-face inter­actions occur with ring centroid-to-centroid distances in the range 5.4–5.7 Å (Fig. 2 ▸ *b*). Inter­actions of the type π–π between rings are also observed with centroid–centroid distances of about 4.17 Å.

## Synthesis and crystallization

A mixture of 5-(4-meth­oxy­phen­yl)-1-phenyl-1*H*-pyrazole-3-carbohydrazide **1** (0.62 g, 2.0 mmol) and isatin **2** (0.30 g, 2.0 mmol) in dry EtOH (15 ml) containing a catalytic qu­antity of concentrated HCl (0.1 ml) was refluxed for 2 h. The mixture was left to cool to 20°C and the orange solid produced was collected by filtration. The product was washed with EtOH, dried, and recrystallized from DMF to give the title compound in 90% yield, m.p. 280–281°C. ^1^H NMR (p.p.m.): 3.72 (*s*, 3H, OMe), 6.89 (*d*, 9.1 Hz, 2H, Ar), 6.92 (*d*, 1H, 7.7 Hz, Ar), 7.08 (*t*, 7.7 Hz, 1H, Ar), 7.15 (*s*, 1H, pyrazol­yl), 7.18 (*d*, 9.1 Hz, 2H, Ar), 7.33–7.36 (*m*, 3H, Ar), 7.44–7.48 (*m*, 3H, Ar), 7.58 (*d*, 7.7 Hz, 1H, Ar), 11.17 (*s*, exch., 1H, NH), 14.08 (*s*, exch., 1H, NH). ^13^C NMR (p.p.m.): 55.7, 108.4, 111.6, 114.7, 120.5, 121.6, 123.1, 126.2, 129.2, 129.3, 129.8, 130.6, 132.2, 138.3, 139.8, 143.1, 145.5, 145.7, 158.4, 160.2, 163.1. Analysis calculated for C_25_H_19_N_5_O_3_ (437.46): C, 68.64; H, 4.38; N, 16.01. Found: C, 68.77; H, 4.47; N, 16.13%.

## Refinement

Crystal data, data collection and structure refinement details are summarized in Table 2 ▸. The meth­oxy­benzene group of one mol­ecule is disordered and was modelled with two components of 0.66 (2) and 0.34 (2) occupancy.

## Supplementary Material

Crystal structure: contains datablock(s) I. DOI: 10.1107/S2414314623004558/hb4430sup1.cif

Structure factors: contains datablock(s) I. DOI: 10.1107/S2414314623004558/hb4430Isup2.hkl

Supporting information file. DOI: 10.1107/S2414314623004558/hb4430Isup3.cml

CCDC reference: 2235858

Additional supporting information:  crystallographic information; 3D view; checkCIF report

## Figures and Tables

**Table 1:** Hydrogen-bond geometry (Å, °)

*D*—H⋯*A*	*D*—H	H⋯*A*	*D*⋯*A*	*D*—H⋯*A*
N1—H1⋯O5^i^	0.86	2.13	2.8594 (16)	142
N3—H3⋯O1	0.86	2.07	2.7419 (17)	135
N6—H6*A*⋯O2	0.86	2.13	2.8512 (17)	142
N8—H8*A*⋯O4	0.86	2.07	2.7396 (16)	134
C5—H5⋯O5^i^	0.93	2.49	3.172 (2)	130
C30—H30⋯O2	0.93	2.52	3.187 (2)	129
C36—H36⋯O3^ii^	0.93	2.44	3.330 (2)	159

Symmetry codes: (i) 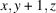 ; (ii) 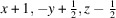 .

**Table 2:** Experimental details

Crystal data	
Chemical formula	C_25_H_19_N_5_O_3_
*M*_r_	437.45
Crystal system, space group	Monoclinic, *P*2_1_/*c*
Temperature (K)	293
*a*, *b*, *c* (Å)	10.8353 (2), 13.3587 (2), 30.3697 (4)
β (°)	91.838 (1)
*V* (Å^3^)	4393.62 (12)
*Z*	8
Radiation type	Cu *K*α
μ (mm^−1^)	0.74
Crystal size (mm)	0.28 × 0.24 × 0.13

Data collection	
Diffractometer	SuperNova, Dual, Cu at home/near, Atlas
Absorption correction	Gaussian (*CrysAlis PRO*; Rigaku OD, 2022 ▸)
*T*_min_, *T*_max_	0.663, 1.000
No. of measured, independent and observed [*I* > 2σ(*I*)] reflections	33362, 8632, 6902
*R*_int_	0.027
(sin θ/λ)_max_ (Å^−1^)	0.620

Refinement	
*R*[*F* ^2^ > 2σ(*F* ^2^)], *wR*(*F* ^2^), *S*	0.042, 0.122, 1.02
No. of reflections	8632
No. of parameters	671
No. of restraints	389
H-atom treatment	H-atom parameters constrained
Δρ_max_, Δρ_min_ (e Å^−3^)	0.16, −0.15

Computer programs: *CrysAlis PRO* 1.171.42.54a (Rigaku OD, 2022 ▸), *SHELXT (Sheldrick, 2015*a* ▸), *SHELXL** (Sheldrick, 2015*b* ▸), *Mercury* (Macrae *et al.*, 2020 ▸) and *ORTEP-3 for Windows* and *WinGX* (Farrugia, 2012 ▸).

## full crystallographic data

### Crystal data

**Table:**

C_25_H_19_N_5_O_3_	*F*(000) = 1824
*M**_r_* = 437.45	*D*_x_ = 1.323 Mg m^−^^3^
Monoclinic, *P*2_1_/*c*	Cu *K*α radiation, λ = 1.54184 Å
*a* = 10.8353 (2) Å	Cell parameters from 14981 reflections
*b* = 13.3587 (2) Å	θ = 4.4–72.8°
*c* = 30.3697 (4) Å	µ = 0.74 mm^−^^1^
β = 91.838 (1)°	*T* = 293 K
*V* = 4393.62 (12) Å^3^	Needle, yellow
*Z* = 8	0.28 × 0.24 × 0.13 mm

### Data collection

**Table:**

SuperNova, Dual, Cu at home/near, Atlas diffractometer	6902 reflections with *I* > 2σ(*I*)
ω scans	*R*_int_ = 0.027
Absorption correction: gaussian (Crysalispro; Rigaku OD, 2022)	θ_max_ = 72.8°, θ_min_ = 3.6°
*T*_min_ = 0.663, *T*_max_ = 1.000	*h* = −11→13
33362 measured reflections	*k* = −16→16
8632 independent reflections	*l* = −37→28

### Refinement

**Table:**

Refinement on *F*^2^	Primary atom site location: dual
Least-squares matrix: full	Hydrogen site location: inferred from neighbouring sites
*R*[*F*^2^ > 2σ(*F*^2^)] = 0.042	H-atom parameters constrained
*wR*(*F*^2^) = 0.122	*w* = 1/[σ^2^(*F*_o_^2^) + (0.0662*P*)^2^ + 0.4435*P*] where *P* = (*F*_o_^2^ + 2*F*_c_^2^)/3
*S* = 1.02	(Δ/σ)_max_ = 0.001
8632 reflections	Δρ_max_ = 0.16 e Å^−^^3^
671 parameters	Δρ_min_ = −0.15 e Å^−^^3^
389 restraints	

### Special details

**Table:**

Geometry. All esds (except the esd in the dihedral angle between two l.s. planes) are estimated using the full covariance matrix. The cell esds are taken into account individually in the estimation of esds in distances, angles and torsion angles; correlations between esds in cell parameters are only used when they are defined by crystal symmetry. An approximate (isotropic) treatment of cell esds is used for estimating esds involving l.s. planes.

### Fractional atomic coordinates and isotropic or equivalent isotropic displacement parameters (Å^2^)

**Table:**

	*x*	*y*	*z*	*U*_iso_*/*U*_eq_	Occ. (<1)
C1	0.52252 (15)	0.83217 (11)	0.32663 (5)	0.0592 (3)	
C2	0.52711 (14)	0.72003 (11)	0.32210 (4)	0.0537 (3)	
C3	0.60695 (14)	0.69953 (11)	0.28547 (5)	0.0555 (3)	
C4	0.64382 (14)	0.79218 (11)	0.26934 (5)	0.0572 (3)	
C5	0.72114 (16)	0.79927 (13)	0.23416 (6)	0.0677 (4)	
H5	0.744764	0.861007	0.223064	0.081*	
C6	0.76180 (18)	0.71073 (14)	0.21621 (6)	0.0742 (5)	
H6	0.815196	0.713327	0.192862	0.089*	
C7	0.72579 (18)	0.61836 (14)	0.23178 (6)	0.0764 (5)	
H7	0.754893	0.560287	0.218787	0.092*	
C8	0.64635 (17)	0.61160 (12)	0.26675 (5)	0.0669 (4)	
H8	0.620651	0.549789	0.277148	0.080*	
C9	0.34347 (14)	0.62106 (11)	0.40400 (5)	0.0550 (3)	
C10	0.28695 (13)	0.66435 (11)	0.44319 (4)	0.0540 (3)	
C11	0.22281 (14)	0.61278 (12)	0.47516 (5)	0.0579 (3)	
H11	0.201499	0.545339	0.475096	0.070*	
C12	0.19783 (14)	0.68269 (11)	0.50672 (5)	0.0568 (3)	
C13	0.14081 (14)	0.66994 (11)	0.54983 (4)	0.0557 (3)	
C14	0.02927 (15)	0.61940 (12)	0.55308 (5)	0.0627 (4)	
H14	−0.011180	0.596060	0.527635	0.075*	
C15	−0.02234 (15)	0.60332 (13)	0.59332 (5)	0.0642 (4)	
H15	−0.097426	0.569987	0.594876	0.077*	
C16	0.03778 (14)	0.63689 (11)	0.63153 (5)	0.0565 (3)	
C17	0.14915 (14)	0.68614 (12)	0.62901 (5)	0.0603 (4)	
H17	0.190144	0.708292	0.654564	0.072*	
C18	0.19988 (14)	0.70257 (12)	0.58833 (5)	0.0609 (4)	
H18	0.274864	0.736086	0.586850	0.073*	
C19	0.0467 (2)	0.63903 (18)	0.71002 (5)	0.0877 (6)	
H19A	0.126011	0.606946	0.709974	0.132*	
H19B	0.001109	0.614749	0.734410	0.132*	
H19C	0.057706	0.710098	0.712737	0.132*	
O3	−0.01949 (11)	0.61720 (10)	0.66988 (3)	0.0730 (3)	
C20	0.24421 (16)	0.86755 (11)	0.51263 (5)	0.0606 (4)	
C21	0.35404 (19)	0.91566 (14)	0.52136 (7)	0.0795 (5)	
H21	0.428034	0.886374	0.513509	0.095*	
C22	0.3541 (2)	1.00840 (16)	0.54202 (8)	0.0942 (6)	
H22	0.428447	1.040920	0.548336	0.113*	
C23	0.2460 (2)	1.05185 (15)	0.55304 (7)	0.0898 (6)	
H23	0.246316	1.114663	0.566242	0.108*	
C24	0.1367 (2)	1.00314 (16)	0.54470 (7)	0.0913 (6)	
H24	0.063160	1.032631	0.552936	0.110*	
C25	0.13386 (19)	0.91034 (15)	0.52413 (6)	0.0787 (5)	
H25	0.059320	0.877735	0.518236	0.094*	
C26	0.54022 (15)	0.33380 (11)	0.32501 (5)	0.0601 (3)	
C27	0.53916 (14)	0.22196 (11)	0.33016 (5)	0.0550 (3)	
C28	0.46467 (15)	0.20092 (11)	0.36808 (5)	0.0586 (3)	
C29	0.42502 (16)	0.29316 (12)	0.38402 (5)	0.0629 (4)	
C30	0.35281 (19)	0.29935 (14)	0.42042 (6)	0.0774 (5)	
H30	0.326915	0.360849	0.431113	0.093*	
C31	0.3203 (2)	0.21097 (15)	0.44043 (6)	0.0851 (6)	
H31	0.271896	0.213253	0.465138	0.102*	
C32	0.3577 (2)	0.11927 (15)	0.42481 (6)	0.0822 (5)	
H32	0.333820	0.061035	0.438997	0.099*	
C33	0.43062 (18)	0.11295 (13)	0.38810 (6)	0.0711 (4)	
H33	0.455702	0.051259	0.377367	0.085*	
C34	0.71499 (14)	0.12317 (11)	0.24600 (5)	0.0549 (3)	
C35	0.79638 (14)	0.16684 (11)	0.21296 (4)	0.0543 (3)	
C36	0.87553 (15)	0.11368 (12)	0.18615 (5)	0.0598 (4)	
H36	0.884597	0.044595	0.184313	0.072*	
C37	0.93705 (14)	0.18535 (11)	0.16310 (5)	0.0565 (3)	
C38	1.0266 (9)	0.1775 (9)	0.1271 (3)	0.0520 (12)	0.66 (2)
C39	1.1282 (9)	0.1166 (8)	0.1344 (3)	0.0658 (14)	0.66 (2)
H39	1.140769	0.086717	0.161813	0.079*	0.66 (2)
C40	1.2120 (8)	0.0992 (7)	0.1015 (3)	0.0681 (13)	0.66 (2)
H40	1.279234	0.057051	0.106735	0.082*	0.66 (2)
C41	1.1947 (6)	0.1448 (5)	0.0612 (2)	0.0566 (11)	0.66 (2)
C42	1.0961 (7)	0.2093 (7)	0.0542 (2)	0.0561 (11)	0.66 (2)
H42	1.086000	0.242043	0.027293	0.067*	0.66 (2)
C43	1.0129 (8)	0.2254 (8)	0.0868 (3)	0.0535 (11)	0.66 (2)
H43	0.946860	0.268871	0.081729	0.064*	0.66 (2)
C44	1.3854 (6)	0.0877 (11)	0.0336 (3)	0.099 (2)	0.66 (2)
H44A	1.432010	0.129168	0.053915	0.148*	0.66 (2)
H44B	1.427862	0.082929	0.006447	0.148*	0.66 (2)
H44C	1.376545	0.022038	0.046014	0.148*	0.66 (2)
O6	1.2671 (7)	0.1303 (5)	0.0256 (2)	0.0755 (11)	0.66 (2)
C38B	1.0365 (18)	0.1668 (18)	0.1326 (6)	0.053 (2)	0.34 (2)
C39B	1.1343 (16)	0.1024 (14)	0.1406 (5)	0.0577 (18)	0.34 (2)
H39B	1.141568	0.070045	0.167671	0.069*	0.34 (2)
C40B	1.2218 (13)	0.0848 (12)	0.1093 (5)	0.0607 (18)	0.34 (2)
H40B	1.289063	0.043364	0.115625	0.073*	0.34 (2)
C41B	1.2078 (11)	0.1296 (9)	0.0685 (4)	0.0589 (18)	0.34 (2)
C42B	1.1093 (14)	0.1924 (12)	0.0595 (5)	0.060 (2)	0.34 (2)
H42B	1.099795	0.221976	0.031910	0.073*	0.34 (2)
C43B	1.0247 (16)	0.2114 (15)	0.0916 (6)	0.058 (2)	0.34 (2)
H43B	0.959046	0.254638	0.085587	0.069*	0.34 (2)
C44B	1.3891 (12)	0.0531 (12)	0.0426 (7)	0.089 (3)	0.34 (2)
H44D	1.436829	0.077974	0.067431	0.134*	0.34 (2)
H44E	1.439382	0.051794	0.017149	0.134*	0.34 (2)
H44F	1.360994	−0.013464	0.048798	0.134*	0.34 (2)
O6B	1.2863 (12)	0.1161 (11)	0.0344 (4)	0.076 (2)	0.34 (2)
C45	0.93673 (15)	0.37295 (11)	0.16561 (5)	0.0583 (4)	
C46	1.06071 (18)	0.39405 (15)	0.17271 (6)	0.0735 (4)	
H46	1.114035	0.345599	0.184366	0.088*	
C47	1.1044 (2)	0.48796 (18)	0.16229 (7)	0.0912 (6)	
H47	1.187712	0.502916	0.166652	0.109*	
C48	1.0248 (2)	0.55904 (16)	0.14551 (8)	0.0948 (7)	
H48	1.054419	0.622241	0.138518	0.114*	
C49	0.9022 (2)	0.53768 (15)	0.13898 (8)	0.0929 (6)	
H49	0.848859	0.586698	0.127869	0.111*	
C50	0.85682 (18)	0.44350 (13)	0.14879 (6)	0.0742 (5)	
H50	0.773622	0.428606	0.144026	0.089*	
N1	0.59191 (13)	0.86917 (9)	0.29427 (4)	0.0644 (3)	
H1	0.602962	0.931952	0.289518	0.077*	
N2	0.47247 (12)	0.65335 (9)	0.34518 (4)	0.0556 (3)	
N3	0.40306 (12)	0.68823 (9)	0.37855 (4)	0.0567 (3)	
H3	0.397058	0.751439	0.383414	0.068*	
N4	0.30144 (12)	0.76075 (10)	0.45310 (4)	0.0589 (3)	
N5	0.24509 (12)	0.77127 (9)	0.49202 (4)	0.0581 (3)	
N6	0.47120 (14)	0.37071 (10)	0.35775 (5)	0.0681 (4)	
H6A	0.457401	0.433331	0.361942	0.082*	
N7	0.59252 (12)	0.15611 (9)	0.30643 (4)	0.0557 (3)	
N8	0.66029 (12)	0.19096 (9)	0.27276 (4)	0.0564 (3)	
H8A	0.668472	0.254206	0.268439	0.068*	
N9	0.80578 (12)	0.26548 (9)	0.20751 (4)	0.0564 (3)	
N10	0.89251 (12)	0.27560 (9)	0.17665 (4)	0.0564 (3)	
O1	0.46685 (13)	0.87878 (8)	0.35461 (4)	0.0761 (3)	
O2	0.34031 (12)	0.53157 (8)	0.39661 (4)	0.0713 (3)	
O4	0.59503 (12)	0.38076 (8)	0.29704 (4)	0.0736 (3)	
O5	0.70216 (12)	0.03351 (8)	0.24977 (4)	0.0735 (3)	

### Atomic displacement parameters (Å^2^)

**Table:**

	*U*^11^	*U*^22^	*U*^33^	*U*^12^	*U*^13^	*U*^23^
C1	0.0688 (9)	0.0552 (8)	0.0545 (8)	−0.0004 (7)	0.0176 (7)	−0.0021 (6)
C2	0.0599 (8)	0.0551 (8)	0.0468 (7)	0.0024 (6)	0.0134 (6)	0.0011 (6)
C3	0.0604 (8)	0.0578 (8)	0.0491 (7)	0.0024 (6)	0.0144 (6)	0.0021 (6)
C4	0.0612 (8)	0.0583 (8)	0.0528 (7)	−0.0007 (6)	0.0140 (6)	−0.0007 (6)
C5	0.0739 (10)	0.0675 (9)	0.0632 (9)	−0.0056 (8)	0.0256 (8)	0.0027 (7)
C6	0.0785 (11)	0.0798 (11)	0.0663 (10)	0.0018 (8)	0.0332 (8)	0.0023 (8)
C7	0.0900 (12)	0.0688 (10)	0.0724 (10)	0.0117 (9)	0.0342 (9)	−0.0025 (8)
C8	0.0804 (10)	0.0588 (9)	0.0632 (9)	0.0068 (7)	0.0265 (8)	0.0026 (7)
C9	0.0601 (8)	0.0550 (8)	0.0506 (7)	0.0054 (6)	0.0152 (6)	0.0013 (6)
C10	0.0600 (8)	0.0565 (8)	0.0465 (7)	0.0052 (6)	0.0149 (6)	0.0011 (6)
C11	0.0662 (8)	0.0589 (8)	0.0495 (7)	−0.0014 (6)	0.0149 (6)	0.0010 (6)
C12	0.0606 (8)	0.0626 (8)	0.0480 (7)	0.0008 (6)	0.0144 (6)	0.0032 (6)
C13	0.0621 (8)	0.0609 (8)	0.0450 (7)	−0.0003 (6)	0.0141 (6)	0.0021 (6)
C14	0.0693 (9)	0.0733 (10)	0.0459 (7)	−0.0107 (7)	0.0078 (6)	−0.0003 (6)
C15	0.0634 (9)	0.0755 (10)	0.0543 (8)	−0.0149 (7)	0.0112 (7)	0.0013 (7)
C16	0.0650 (8)	0.0597 (8)	0.0460 (7)	−0.0032 (6)	0.0169 (6)	0.0005 (6)
C17	0.0667 (9)	0.0688 (9)	0.0459 (7)	−0.0077 (7)	0.0083 (6)	−0.0030 (6)
C18	0.0574 (8)	0.0728 (9)	0.0532 (8)	−0.0082 (7)	0.0121 (6)	0.0008 (7)
C19	0.0961 (13)	0.1231 (16)	0.0450 (8)	−0.0197 (12)	0.0185 (8)	−0.0056 (9)
O3	0.0809 (7)	0.0924 (8)	0.0469 (5)	−0.0197 (6)	0.0211 (5)	−0.0014 (5)
C20	0.0778 (10)	0.0571 (8)	0.0480 (7)	0.0049 (7)	0.0183 (7)	0.0017 (6)
C21	0.0833 (12)	0.0680 (10)	0.0885 (12)	−0.0030 (9)	0.0238 (10)	−0.0084 (9)
C22	0.1095 (16)	0.0710 (12)	0.1028 (16)	−0.0114 (11)	0.0159 (12)	−0.0112 (11)
C23	0.1319 (19)	0.0631 (10)	0.0750 (12)	0.0113 (12)	0.0143 (12)	−0.0078 (9)
C24	0.1104 (16)	0.0816 (13)	0.0831 (13)	0.0319 (12)	0.0216 (11)	−0.0075 (10)
C25	0.0813 (11)	0.0792 (11)	0.0764 (11)	0.0142 (9)	0.0147 (9)	−0.0068 (9)
C26	0.0657 (9)	0.0563 (8)	0.0591 (8)	0.0036 (7)	0.0163 (7)	0.0006 (6)
C27	0.0606 (8)	0.0555 (8)	0.0497 (7)	0.0027 (6)	0.0153 (6)	0.0009 (6)
C28	0.0672 (9)	0.0594 (8)	0.0503 (7)	0.0046 (7)	0.0167 (6)	−0.0010 (6)
C29	0.0697 (9)	0.0633 (9)	0.0567 (8)	0.0074 (7)	0.0176 (7)	−0.0012 (7)
C30	0.0917 (12)	0.0751 (11)	0.0672 (10)	0.0139 (9)	0.0314 (9)	−0.0039 (8)
C31	0.1029 (14)	0.0889 (13)	0.0661 (10)	0.0103 (10)	0.0427 (10)	0.0021 (9)
C32	0.1050 (14)	0.0753 (11)	0.0687 (10)	0.0029 (10)	0.0392 (10)	0.0087 (8)
C33	0.0886 (11)	0.0635 (9)	0.0629 (9)	0.0047 (8)	0.0290 (8)	0.0023 (7)
C34	0.0640 (8)	0.0540 (8)	0.0476 (7)	−0.0008 (6)	0.0162 (6)	0.0017 (6)
C35	0.0627 (8)	0.0551 (8)	0.0461 (7)	−0.0029 (6)	0.0160 (6)	−0.0005 (6)
C36	0.0701 (9)	0.0557 (8)	0.0549 (8)	0.0015 (7)	0.0214 (7)	0.0000 (6)
C37	0.0616 (8)	0.0604 (8)	0.0483 (7)	−0.0016 (6)	0.0151 (6)	−0.0018 (6)
C38	0.056 (2)	0.056 (3)	0.046 (3)	−0.0001 (14)	0.0142 (19)	0.0002 (19)
C39	0.073 (2)	0.074 (3)	0.050 (2)	0.009 (2)	0.0096 (17)	0.0135 (17)
C40	0.0618 (19)	0.078 (3)	0.065 (3)	0.0170 (17)	0.0098 (18)	0.0058 (19)
C41	0.0548 (19)	0.067 (2)	0.0491 (18)	−0.0030 (16)	0.0123 (14)	−0.0025 (15)
C42	0.0629 (19)	0.061 (2)	0.0455 (19)	−0.0002 (14)	0.0121 (14)	0.0042 (15)
C43	0.057 (2)	0.057 (3)	0.0468 (19)	0.0022 (14)	0.0097 (15)	0.0048 (15)
C44	0.066 (2)	0.150 (6)	0.081 (3)	0.018 (3)	0.0134 (19)	−0.015 (4)
O6	0.062 (2)	0.107 (2)	0.059 (2)	0.0102 (14)	0.0207 (15)	−0.0021 (15)
C38B	0.058 (3)	0.057 (4)	0.045 (3)	−0.003 (3)	0.009 (3)	0.004 (3)
C39B	0.059 (3)	0.062 (4)	0.053 (4)	0.008 (2)	0.014 (3)	0.010 (3)
C40B	0.054 (3)	0.068 (4)	0.061 (3)	0.005 (3)	0.011 (3)	0.004 (3)
C41B	0.054 (3)	0.068 (3)	0.055 (3)	0.004 (2)	0.017 (3)	−0.003 (3)
C42B	0.072 (4)	0.064 (4)	0.046 (3)	0.001 (3)	0.015 (3)	0.007 (3)
C43B	0.060 (3)	0.059 (4)	0.055 (4)	0.010 (3)	0.010 (3)	0.000 (3)
C44B	0.065 (4)	0.113 (7)	0.090 (6)	0.019 (4)	0.030 (4)	−0.017 (5)
O6B	0.063 (4)	0.105 (4)	0.062 (4)	0.013 (3)	0.026 (3)	−0.005 (3)
C45	0.0724 (9)	0.0571 (8)	0.0467 (7)	−0.0090 (7)	0.0226 (6)	−0.0036 (6)
C46	0.0792 (11)	0.0818 (11)	0.0599 (9)	−0.0162 (9)	0.0080 (8)	−0.0003 (8)
C47	0.0998 (15)	0.0941 (15)	0.0808 (12)	−0.0401 (12)	0.0173 (11)	−0.0070 (11)
C48	0.1280 (19)	0.0671 (11)	0.0918 (14)	−0.0281 (12)	0.0423 (13)	−0.0041 (10)
C49	0.1182 (17)	0.0608 (10)	0.1020 (15)	0.0036 (11)	0.0387 (13)	0.0094 (10)
C50	0.0791 (11)	0.0633 (9)	0.0819 (11)	0.0010 (8)	0.0278 (9)	0.0026 (8)
N1	0.0813 (9)	0.0516 (7)	0.0619 (7)	−0.0047 (6)	0.0264 (6)	−0.0014 (5)
N2	0.0647 (7)	0.0553 (6)	0.0479 (6)	0.0042 (5)	0.0181 (5)	0.0000 (5)
N3	0.0703 (7)	0.0520 (6)	0.0490 (6)	0.0028 (5)	0.0217 (5)	0.0004 (5)
N4	0.0690 (7)	0.0611 (7)	0.0478 (6)	0.0031 (6)	0.0217 (5)	0.0010 (5)
N5	0.0697 (7)	0.0581 (7)	0.0477 (6)	0.0011 (6)	0.0205 (5)	0.0000 (5)
N6	0.0822 (9)	0.0546 (7)	0.0692 (8)	0.0079 (6)	0.0271 (7)	−0.0019 (6)
N7	0.0645 (7)	0.0559 (7)	0.0477 (6)	0.0004 (5)	0.0186 (5)	0.0017 (5)
N8	0.0676 (7)	0.0516 (6)	0.0513 (6)	−0.0004 (5)	0.0228 (5)	0.0024 (5)
N9	0.0650 (7)	0.0571 (7)	0.0484 (6)	−0.0046 (5)	0.0212 (5)	−0.0006 (5)
N10	0.0656 (7)	0.0553 (7)	0.0496 (6)	−0.0053 (5)	0.0206 (5)	−0.0016 (5)
O1	0.1001 (9)	0.0573 (6)	0.0732 (7)	0.0007 (6)	0.0383 (6)	−0.0079 (5)
O2	0.0912 (8)	0.0544 (6)	0.0703 (7)	−0.0001 (5)	0.0347 (6)	−0.0019 (5)
O4	0.0886 (8)	0.0566 (6)	0.0777 (7)	0.0020 (5)	0.0337 (6)	0.0079 (5)
O5	0.1010 (9)	0.0524 (6)	0.0696 (7)	−0.0023 (5)	0.0404 (6)	0.0010 (5)

### Geometric parameters (Å, º)

**Table:**

C1—O1	1.2275 (18)	C31—H31	0.9300
C1—N1	1.3500 (19)	C32—C33	1.390 (2)
C1—C2	1.505 (2)	C32—H32	0.9300
C2—N2	1.2892 (18)	C33—H33	0.9300
C2—C3	1.4568 (18)	C34—O5	1.2117 (18)
C3—C8	1.379 (2)	C34—N8	1.3650 (18)
C3—C4	1.394 (2)	C34—C35	1.4771 (18)
C4—C5	1.382 (2)	C35—N9	1.3323 (19)
C4—N1	1.4053 (19)	C35—C36	1.3955 (19)
C5—C6	1.380 (2)	C36—C37	1.371 (2)
C5—H5	0.9300	C36—H36	0.9300
C6—C7	1.382 (3)	C37—N10	1.3670 (19)
C6—H6	0.9300	C37—C38B	1.464 (14)
C7—C8	1.391 (2)	C37—C38	1.489 (7)
C7—H7	0.9300	C38—C39	1.380 (4)
C8—H8	0.9300	C38—C43	1.384 (4)
C9—O2	1.2166 (18)	C39—C40	1.391 (4)
C9—N3	1.3607 (18)	C39—H39	0.9300
C9—C10	1.4741 (18)	C40—C41	1.374 (4)
C10—N4	1.331 (2)	C40—H40	0.9300
C10—C11	1.3941 (19)	C41—O6	1.372 (4)
C11—C12	1.371 (2)	C41—C42	1.384 (4)
C11—H11	0.9300	C42—C43	1.378 (4)
C12—N5	1.3698 (19)	C42—H42	0.9300
C12—C13	1.4754 (18)	C43—H43	0.9300
C13—C18	1.385 (2)	C44—O6	1.416 (5)
C13—C14	1.391 (2)	C44—H44A	0.9600
C14—C15	1.377 (2)	C44—H44B	0.9600
C14—H14	0.9300	C44—H44C	0.9600
C15—C16	1.387 (2)	C38B—C39B	1.380 (7)
C15—H15	0.9300	C38B—C43B	1.382 (7)
C16—O3	1.3627 (16)	C39B—C40B	1.385 (6)
C16—C17	1.379 (2)	C39B—H39B	0.9300
C17—C18	1.3858 (19)	C40B—C41B	1.378 (7)
C17—H17	0.9300	C40B—H40B	0.9300
C18—H18	0.9300	C41B—O6B	1.373 (6)
C19—O3	1.425 (2)	C41B—C42B	1.378 (7)
C19—H19A	0.9600	C42B—C43B	1.384 (6)
C19—H19B	0.9600	C42B—H42B	0.9300
C19—H19C	0.9600	C43B—H43B	0.9300
C20—C21	1.371 (3)	C44B—O6B	1.412 (7)
C20—C25	1.380 (2)	C44B—H44D	0.9600
C20—N5	1.4305 (19)	C44B—H44E	0.9600
C21—C22	1.389 (3)	C44B—H44F	0.9600
C21—H21	0.9300	C45—C50	1.368 (3)
C22—C23	1.359 (3)	C45—C46	1.383 (2)
C22—H22	0.9300	C45—N10	1.4294 (18)
C23—C24	1.368 (3)	C46—C47	1.381 (3)
C23—H23	0.9300	C46—H46	0.9300
C24—C25	1.388 (3)	C47—C48	1.370 (3)
C24—H24	0.9300	C47—H47	0.9300
C25—H25	0.9300	C48—C49	1.367 (3)
C26—O4	1.2249 (18)	C48—H48	0.9300
C26—N6	1.3559 (19)	C49—C50	1.387 (3)
C26—C27	1.502 (2)	C49—H49	0.9300
C27—N7	1.2862 (18)	C50—H50	0.9300
C27—C28	1.4545 (19)	N1—H1	0.8600
C28—C33	1.379 (2)	N2—N3	1.3633 (15)
C28—C29	1.397 (2)	N3—H3	0.8600
C29—C30	1.377 (2)	N4—N5	1.3551 (15)
C29—N6	1.409 (2)	N6—H6A	0.8600
C30—C31	1.378 (3)	N7—N8	1.3600 (15)
C30—H30	0.9300	N8—H8A	0.8600
C31—C32	1.379 (3)	N9—N10	1.3552 (15)
			
O1—C1—N1	128.01 (14)	O5—C34—C35	121.83 (13)
O1—C1—C2	125.91 (13)	N8—C34—C35	115.00 (12)
N1—C1—C2	106.09 (12)	N9—C35—C36	112.34 (12)
N2—C2—C3	125.42 (13)	N9—C35—C34	121.64 (12)
N2—C2—C1	128.38 (12)	C36—C35—C34	125.93 (13)
C3—C2—C1	106.20 (12)	C37—C36—C35	105.10 (13)
C8—C3—C4	121.01 (13)	C37—C36—H36	127.5
C8—C3—C2	132.41 (14)	C35—C36—H36	127.5
C4—C3—C2	106.58 (12)	N10—C37—C36	106.26 (12)
C5—C4—C3	121.34 (14)	N10—C37—C38B	127.9 (10)
C5—C4—N1	129.02 (14)	C36—C37—C38B	125.7 (9)
C3—C4—N1	109.64 (12)	N10—C37—C38	121.9 (5)
C6—C5—C4	117.12 (15)	C36—C37—C38	131.6 (5)
C6—C5—H5	121.4	C39—C38—C43	118.5 (4)
C4—C5—H5	121.4	C39—C38—C37	117.3 (5)
C5—C6—C7	122.18 (15)	C43—C38—C37	124.2 (5)
C5—C6—H6	118.9	C38—C39—C40	121.3 (4)
C7—C6—H6	118.9	C38—C39—H39	119.4
C6—C7—C8	120.49 (16)	C40—C39—H39	119.4
C6—C7—H7	119.8	C41—C40—C39	119.4 (4)
C8—C7—H7	119.8	C41—C40—H40	120.3
C3—C8—C7	117.84 (15)	C39—C40—H40	120.3
C3—C8—H8	121.1	O6—C41—C40	125.1 (4)
C7—C8—H8	121.1	O6—C41—C42	115.1 (4)
O2—C9—N3	123.67 (13)	C40—C41—C42	119.8 (4)
O2—C9—C10	121.62 (13)	C43—C42—C41	120.4 (4)
N3—C9—C10	114.64 (12)	C43—C42—H42	119.8
N4—C10—C11	112.24 (12)	C41—C42—H42	119.8
N4—C10—C9	120.88 (12)	C42—C43—C38	120.6 (4)
C11—C10—C9	126.71 (14)	C42—C43—H43	119.7
C12—C11—C10	105.32 (13)	C38—C43—H43	119.7
C12—C11—H11	127.3	O6—C44—H44A	109.5
C10—C11—H11	127.3	O6—C44—H44B	109.5
N5—C12—C11	106.04 (12)	H44A—C44—H44B	109.5
N5—C12—C13	124.09 (13)	O6—C44—H44C	109.5
C11—C12—C13	129.74 (14)	H44A—C44—H44C	109.5
C18—C13—C14	118.16 (13)	H44B—C44—H44C	109.5
C18—C13—C12	121.16 (13)	C41—O6—C44	117.2 (4)
C14—C13—C12	120.57 (13)	C39B—C38B—C43B	118.4 (8)
C15—C14—C13	121.15 (14)	C39B—C38B—C37	124.9 (10)
C15—C14—H14	119.4	C43B—C38B—C37	116.6 (10)
C13—C14—H14	119.4	C38B—C39B—C40B	121.6 (8)
C14—C15—C16	119.92 (14)	C38B—C39B—H39B	119.2
C14—C15—H15	120.0	C40B—C39B—H39B	119.2
C16—C15—H15	120.0	C41B—C40B—C39B	119.0 (7)
O3—C16—C17	124.25 (14)	C41B—C40B—H40B	120.5
O3—C16—C15	115.98 (13)	C39B—C40B—H40B	120.5
C17—C16—C15	119.77 (13)	O6B—C41B—C40B	124.6 (7)
C16—C17—C18	119.86 (14)	O6B—C41B—C42B	115.1 (7)
C16—C17—H17	120.1	C40B—C41B—C42B	120.3 (7)
C18—C17—H17	120.1	C41B—C42B—C43B	119.9 (7)
C13—C18—C17	121.13 (14)	C41B—C42B—H42B	120.1
C13—C18—H18	119.4	C43B—C42B—H42B	120.1
C17—C18—H18	119.4	C38B—C43B—C42B	120.7 (8)
O3—C19—H19A	109.5	C38B—C43B—H43B	119.6
O3—C19—H19B	109.5	C42B—C43B—H43B	119.6
H19A—C19—H19B	109.5	O6B—C44B—H44D	109.5
O3—C19—H19C	109.5	O6B—C44B—H44E	109.5
H19A—C19—H19C	109.5	H44D—C44B—H44E	109.5
H19B—C19—H19C	109.5	O6B—C44B—H44F	109.5
C16—O3—C19	117.47 (13)	H44D—C44B—H44F	109.5
C21—C20—C25	120.70 (16)	H44E—C44B—H44F	109.5
C21—C20—N5	119.22 (15)	C41B—O6B—C44B	116.7 (8)
C25—C20—N5	120.07 (16)	C50—C45—C46	121.20 (16)
C20—C21—C22	119.56 (19)	C50—C45—N10	120.11 (15)
C20—C21—H21	120.2	C46—C45—N10	118.69 (15)
C22—C21—H21	120.2	C47—C46—C45	119.2 (2)
C23—C22—C21	120.3 (2)	C47—C46—H46	120.4
C23—C22—H22	119.8	C45—C46—H46	120.4
C21—C22—H22	119.8	C48—C47—C46	119.9 (2)
C22—C23—C24	119.96 (19)	C48—C47—H47	120.0
C22—C23—H23	120.0	C46—C47—H47	120.0
C24—C23—H23	120.0	C49—C48—C47	120.41 (19)
C23—C24—C25	121.0 (2)	C49—C48—H48	119.8
C23—C24—H24	119.5	C47—C48—H48	119.8
C25—C24—H24	119.5	C48—C49—C50	120.5 (2)
C20—C25—C24	118.5 (2)	C48—C49—H49	119.7
C20—C25—H25	120.8	C50—C49—H49	119.7
C24—C25—H25	120.8	C45—C50—C49	118.73 (19)
O4—C26—N6	127.84 (15)	C45—C50—H50	120.6
O4—C26—C27	125.95 (13)	C49—C50—H50	120.6
N6—C26—C27	106.20 (12)	C1—N1—C4	111.48 (12)
N7—C27—C28	125.61 (13)	C1—N1—H1	124.3
N7—C27—C26	128.08 (13)	C4—N1—H1	124.3
C28—C27—C26	106.31 (12)	C2—N2—N3	116.22 (12)
C33—C28—C29	120.62 (14)	C9—N3—N2	118.68 (12)
C33—C28—C27	132.60 (14)	C9—N3—H3	120.7
C29—C28—C27	106.77 (13)	N2—N3—H3	120.7
C30—C29—C28	121.35 (15)	C10—N4—N5	104.15 (11)
C30—C29—N6	129.16 (15)	N4—N5—C12	112.22 (12)
C28—C29—N6	109.49 (13)	N4—N5—C20	118.97 (12)
C29—C30—C31	117.51 (16)	C12—N5—C20	128.78 (11)
C29—C30—H30	121.2	C26—N6—C29	111.22 (13)
C31—C30—H30	121.2	C26—N6—H6A	124.4
C30—C31—C32	121.83 (15)	C29—N6—H6A	124.4
C30—C31—H31	119.1	C27—N7—N8	116.77 (12)
C32—C31—H31	119.1	N7—N8—C34	118.41 (12)
C31—C32—C33	120.71 (17)	N7—N8—H8A	120.8
C31—C32—H32	119.6	C34—N8—H8A	120.8
C33—C32—H32	119.6	C35—N9—N10	104.00 (11)
C28—C33—C32	117.98 (16)	N9—N10—C37	112.30 (11)
C28—C33—H33	121.0	N9—N10—C45	119.87 (11)
C32—C33—H33	121.0	C37—N10—C45	127.42 (11)
O5—C34—N8	123.11 (13)		

### Hydrogen-bond geometry (Å, º)

**Table:**

*D*—H···*A*	*D*—H	H···*A*	*D*···*A*	*D*—H···*A*
N1—H1···O5^i^	0.86	2.13	2.8594 (16)	142
N3—H3···O1	0.86	2.07	2.7419 (17)	135
N6—H6*A*···O2	0.86	2.13	2.8512 (17)	142
N8—H8*A*···O4	0.86	2.07	2.7396 (16)	134
C5—H5···O5^i^	0.93	2.49	3.172 (2)	130
C30—H30···O2	0.93	2.52	3.187 (2)	129
C36—H36···O3^ii^	0.93	2.44	3.330 (2)	159

Symmetry codes: (i) *x*, *y*+1, *z*; (ii) *x*+1, −*y*+1/2, *z*−1/2.

## References

[bb1] Afsah, E. M., Elmorsy, S. S., Abdelmageed, S. M. & Zaki, Z. E. (2016). *Z. Naturforsch. B*, **71**, 1147–1157.

[bb17] Farrugia, L. J. (2012). *J. Appl. Cryst.* **45**, 849–854.

[bb2] Kariuki, B. M., Abdel-Wahab, B. F., Farahat, A. A. & El-Hiti, G. A. (2022). *Molbank*, **2022**, M1374.

[bb3] Karrouchi, K., Radi, S., Ramli, Y., Taoufik, J., Mabkhot, Y. N., Al-aizari, F. A. & Ansar, M. (2018). *Molecules*, **23**, 134131. 10.3390/molecules23010134 PMC6017056 29329257

[bb4] Kaur, M., Singh, M., Chadha, N. & Silakari, O. (2016). *Eur. J. Med. Chem.* **123**, 858–894. 10.1016/j.ejmech.2016.08.011 27543880

[bb5] Li, X., Yu, Y. & Tu, Z. (2021). *Molecules*, **26**, 1202. 10.3390/molecules26051202 PMC7956461 33668128

[bb6] Lu, X.-Y., Wang, Z.-C., Wei, T., Yan, X.-Q., Wang, P.-F. & Zhu, H.-L. (2016). *RSC Adv.* **6**, 22917–22935.

[bb18] Macrae, C. F., Sovago, I., Cottrell, S. J., Galek, P. T. A., McCabe, P., Pidcock, E., Platings, M., Shields, G. P., Stevens, J. S., Towler, M. & Wood, P. A. (2020). *J. Appl. Cryst.* **53**, 226–235. 10.1107/S1600576719014092 PMC6998782 32047413

[bb7] Mohamed, H. A., Bekheit, M. S., Ewies, E. F., Awad, H. M., Betz, R., Hosten, E. C. & Abdel-Wahab, B. F. (2023). *J. Mol. Struct.* **1274**, 134415.

[bb8] Qian, P., Su, J.-H., Wang, Y., Bi, M., Zha, Z. & Wang, Z. (2017). *J. Org. Chem.* **82**, 6434–6440. 10.1021/acs.joc.7b00635 28535683

[bb9] Rigaku OD. (2022). *CrysAlis PRO*. Rigaku Oxford Diffraction, Oxford, England.

[bb10] Ríos, M.-C. & Portilla, J. (2022). *Chemistry*, **4**, 940–968.

[bb11] Sheldrick, G. M. (2015*a*). *Acta Cryst.* A**71**, 3–8.

[bb12] Sheldrick, G. M. (2015*b*). *Acta Cryst.* C**71**, 3–8.

[bb13] Wei, W.-T., Ying, W.-W., Zhu, W.-M., Wu, Y., Huang, Y.-L., Cao, Y.-Q., Wang, Y.-N. & Liang, H. (2017). *Synlett*, **28**, 2307–2310.

[bb14] Ying, W.-W., Zhu, W.-M., Liang, H. & Wei, W.-T. (2018). *Synlett*, **29**, 215–218.

[bb15] Zi, Y., Cai, Z.-J., Wang, S.-Y. & Ji, S.-J. (2014). *Org. Lett.* **16**, 3094–3097. 10.1021/ol501203q 24850466

[bb16] Zora, M., Kivrak, A. & Yazici, C. (2011). *J. Org. Chem.* **76**, 6726–6742. 10.1021/jo201119e 21739980

